# The Effect of Different Acupuncture Therapies on Neurological Recovery in Spinal Cord Injury: A Systematic Review and Network Meta-Analysis of Randomized Controlled Trials

**DOI:** 10.1155/2019/2371084

**Published:** 2019-10-27

**Authors:** Feng Xiong, Chenying Fu, Qing Zhang, Lihong Peng, Zejun Liang, Li Chen, Chengqi He, Quan Wei

**Affiliations:** ^1^Department of Rehabilitation Medicine Center, West China Hospital, Sichuan University, Chengdu, Sichuan, China; ^2^Key Laboratory of Rehabilitation Medicine in Sichuan Province, Chengdu, Sichuan, China; ^3^State Key Laboratory of Biotherapy, West China Hospital, Sichuan University, Chengdu, Sichuan, China

## Abstract

Many acupuncture therapies were used to treat spinal cord injury (SCI) and its complications. The difference in efficacy among these therapies has not been assessed. To compare the efficacy of different acupuncture therapies for SCI, we searched databases (PubMed, Embase, Cochrane Library, CNKI, and WanFang) for relevant RCTs in both English and Chinese before June 2019 that reported the association between acupuncture therapies and SCI. The RCTs were categorized according to the location of the acupoints used in them. The neural function was assessed by American Spinal Injury Association (ASIA) motor score, and daily living ability was accessed by Modified Barthel Index (MBI) after SCI. In total, 22 trials involving 1644 participants were included. The pairwise meta-analysis and random effects model network meta-analysis were conducted. The results indicated that exercise combined with electro-acupuncture (EA) is superior to exercise without acupuncture in improving the ASIA motor score. EA was associated with a significantly higher improvement in the MBI score than exercise alone, except for EA of head + limbs and limbs. Additionally, EA on the head + back and back + front (chest and abdomen) rank the top in both increasing the ASIA motor score and the MBI score. Acupuncture can significantly increase motor function and daily living ability of individuals who suffer from SCI, especially acupuncture of the back + front or the head + back. The evidence supports acupuncture of the back + front or the head + back as an effective treatment for SCI.

## 1. Introduction

SCI and its secondary complications have always been a great social and economic burden for the patients' families and health care system. It is estimated that the annual occurrence of SCI is roughly 17,000 new cases each year or approximately 54 cases per million population in the US. Take patient with high tetraplegia (C1–C4), for example, the average yearly expenses (heath care and living expenses) cost an average of 1,065,980 dollars in the first year and 185,111 dollars in the each subsequent year which did not even include any indirect costs such as losses in wages, fringe benefits, and productivity which cost average 72,047 dollars per year in 2015 in the US [1].

People with SCI almost always show some degree of motor and sensory function recovery below the injury level, but spontaneous recovery in patients with complete SCI is quite limited. [2] Although there have been reports of positive outcomes following treatment options such as surgery [3], pharmacological interventions [4], rehabilitation [5], and alternative methods such as acupuncture [6–9], reviews by a number of researchers concluded that use of acupuncture or EA in acute SCI can significantly improve functional recovery in long-term and help the management of chronic pain associated with these injuries, with a risk that could be considered minimum [6, 7, 10–12].

At present, a standard acupuncture therapy for people with SCI is not yet available. So, the question remains that which one of these existing therapies is most effective? Thus, a comparison of efficacy of different acupuncture therapies is necessary.

However, a meta-analysis of direct comparisons between different acupuncture therapies is unavailable because such analyses are limited by comparators and insufficient studies which probably due to the fact that acupuncture is not yet widely used throughout the world.

Network meta-analysis overcomes this limitation by creating indirect comparisons and allowing data synthesis, which could help identify the most effective interventions. Therefore, we performed this Bayesian network meta-analysis to compare efficacy of the different acupuncture therapies, which include both direct and indirect comparisons of the changes in both the ASIA motor score and the MBI score of these methods.

## 2. Methods

This study was conducted following the Preferred Reporting Items for Systematic Reviews and Meta-Analyses for Network Meta-Analysis (PRISMA-NMA) checklist [13].

### 2.1. Study Selection

Two authors (Feng Xiong and Chenying Fu) independently identified the eligible studies, firstly by titles and abstracts, and then full texts were scanned. Any disagreements regarding study inclusion were resolved by discussion with a third author (Lihong Peng or Zejun Liang).

### 2.2. Eligibility Criteria

Randomized controlled trials that met following criteria were included: (1) participants diagnosed with SCI; (2) trials comparing different acupuncture/EA therapies, or comparing acupuncture/EA to regular rehabilitation training or drugs; (3) trials reporting one of following outcomes: ASIA motor score and MBI score.

The inclusion of studies was limited to RCTs reporting the prospective use of the acupuncture interventions in study participants who suffered from spinal cord injury (SCI). All trials selected were those that focused on functional recovery, while those that only reported complications such as bladder dysfunction or pain were excluded. This review included parallel-group RCTs that assessed the efficacy of acupuncture regardless of blinding or the type of control comparison.

Our study mainly considered needle acupuncture as the intervention of interest which included manual acupuncture, electro-acupuncture, auricular acupuncture, and acupoint injection. Laser acupuncture, acupressure, and moxibustion were excluded. Studies on complications, prospective nonrandomized studies, duplicate publications, or studies without extractable data for analysis were also excluded.

### 2.3. Data Sources and Searches

**Figure 1:**
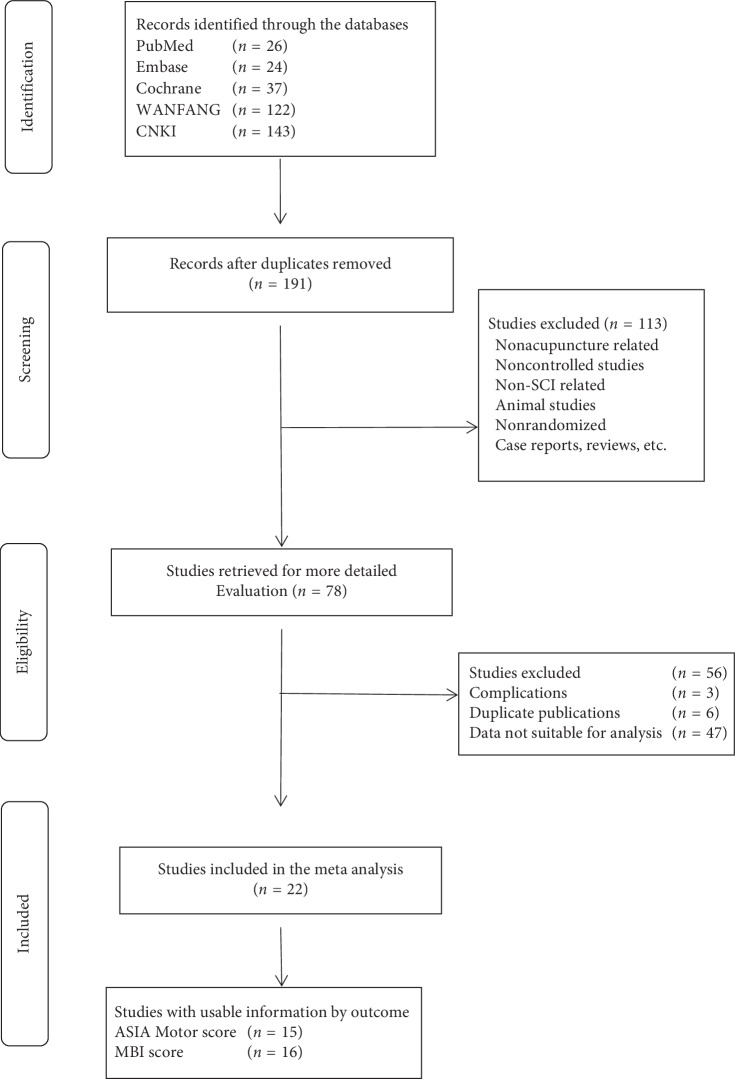
PRISMA flowchart of the study selection process.

We performed searches of the following databases: PubMed, Embase, Cochrane Library, CNKI, and WanFang database. The search keywords included SCI patients, acupuncture, EA, and randomized controlled clinical trials. The following keywords were used in combination with both MeSH terms and text words: SCI, acupuncture, EA, and randomized controlled trials. No restriction was placed on publication status or language. In addition, to guarantee the saturation of literature, we also scanned relevant trials included by previous systematic reviews and meta-analyses for publication as well. The study selection process is shown in Figure 1.

### 2.4. Data Collection Process

Four authors extracted and assessed the data (data extraction: Feng Xiong and Chenying Fu; assessment: Zejun Liang and Lihong Peng) using a standard spread sheet (performed by Excel; Version 2010) consisting of four sheets, which included (1) general information (i.e., study design, intervention types, and information about data extractor); (2) study characteristic (i.e., patients, intervention, frequency, duration, follow-up, and outcomes); (3) a risk of bias assessment (ROB: randomization, allocation, blinding of participants and personnel, blinding of outcome assessor, incomplete data, and selective reporting); and (4) a summary of outcome data (ASIA motor score and MBI score).

### 2.5. Categorizing Strategy

Acupuncturists use different combinations of acupoints to treat different conditions, according to the function of each meridian and acupoint. Since up to 20 meridians are used in acupuncture (acupoints used in ear acupuncture not included), theoretically there can be 1,048,576 kinds of meridian combinations. If we judge the therapies in combinations of acupoints, the number would only be greater. It would be impossible to compare all those different combinations this way. So, we settled for comparing combinations of four main regions. The meridians in the same region are thought to be interconnected and functionally related. As for the scalp and ear acupuncture, it would be ideal to classify them as different therapies, but due to limited number of RCTs, they were classified into the same group, the head acupuncture.

The regions include the back (governor meridian, Jiaji acupoints, bladder meridian, and Yangming meridian), the front (Ren channel, kidney meridian, and stomach meridian), the head (scalp acupoints, ear acupoints), and the limb (three yin meridians of hand, three yin meridians of foot, three yang meridians of hand, and three yang meridians of foot). In this way, each combination would have more RCTs to compare with. The included studies each used one or a combination of these regions for acupuncture in treating SCI patients and many produced positive outcomes.

For example, some studies may only use acupoints from the back and the head (back + head), and some others may only use acupoints from the back and the limb (back + limb).

### 2.6. Statistical Analysis

**Figure 2:**
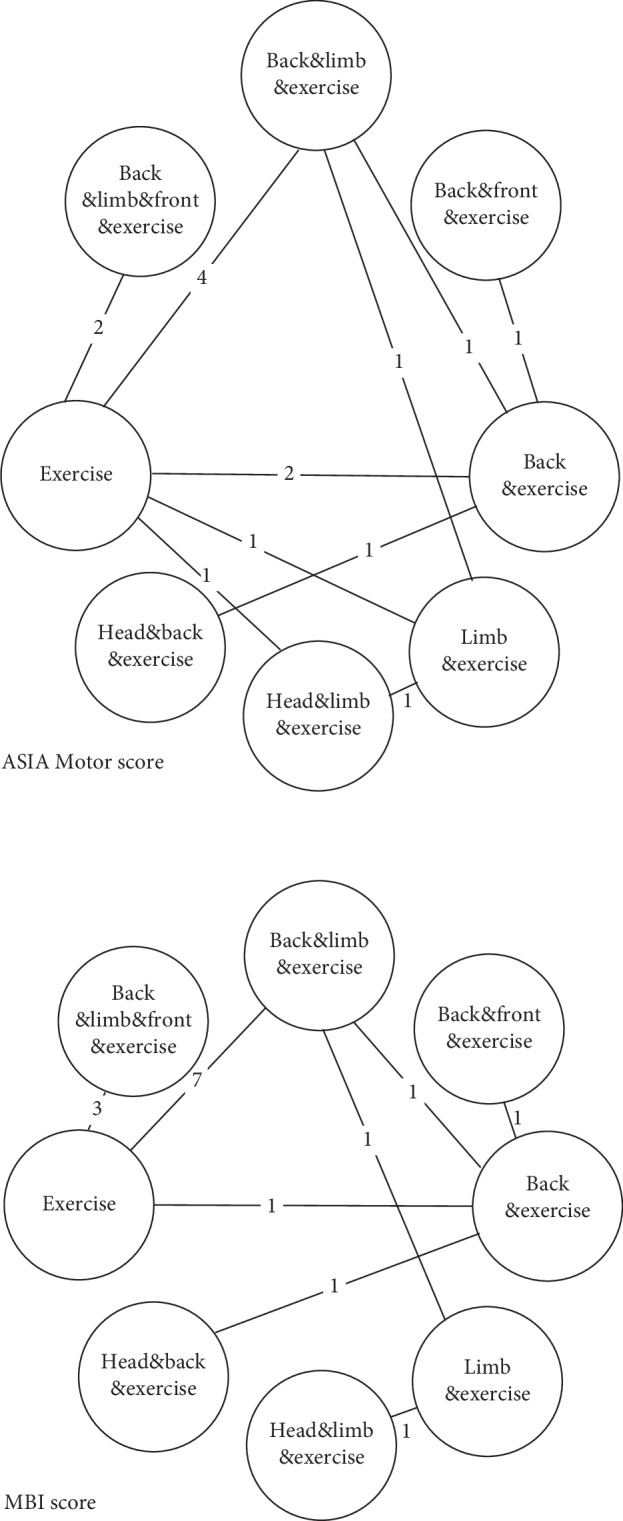
Network plot of different therapies by ASIA and MBI scale.

The pairwise meta-analysis was performed to synthesize studies that compared the same interventions with random effect models (direct comparison) using the R software (version 3.5.1; http://www.r-project.org). Then, to determine comparative effectiveness, a random effects model network meta-analysis (a combination of direct and indirect comparison) was conducted in a Bayesian framework by introducing the WinBUGS package and the Jags package (http://www.r-project.org). The direct and indirect comparisons for each specific pair of treatments were combined by modeling the outcomes in every treatment group of included studies. The ASIA motor and MBI score were reported as a standard mean difference (SMD) with 95% confidence intervals (CI) for direct comparisons or 95% credible intervals (CrI) for indirect comparisons. The Brooks–Gelman–Rubin method was then used to assess the convergence between direct and indirect variances. To show the relationship between different acupuncture treatments, we used network plots to show direct comparisons between arms (see Figure 2). The effectiveness of each treatment among all existing treatments was ranked in order by calculating the SMD; probability density functions were generated. The *I*^2^ test was conducted to quantitatively assess heterogeneity, and an *I*^2^ value greater than 75% was considered significant.

The clinical, statistical, and methodological differences may attribute to heterogeneity. The statistical codes were shown in Supplement 1 ([Supplementary-material supplementary-material-1]).

## 3. Results

### 3.1. Study Selection

The search was performed and updated up to date (June 5th, 2019), and 191 references were identified. After duplicate studies were removed, other 113 records were excluded by reading the titles and abstracts, and the full texts of 78 articles were then assessed for eligibility. A total of 22 studies were selected covering 8 groups which include exercise, 7 different combinations of exercise, and acupuncture of different regions (the back, the limbs, the head, and the front).

### 3.2. Study Description

Overall, 1644 patients were included in the network meta-analysis. The characteristics of included studies are summarized in Table 1. It should be noted that some of the included studies did not emphasize that they were RCTs in the title, even when in fact they were. All studies reported ASIA motor scale and/or MBI score as an outcome. Out of 22 studies, 15 reported ASIA motor scale as an outcome [12, 14–27], and 16 reported MBI as an outcome [19–34]. The network plot of eligible comparisons for the ASIA motor scale is shown in Figure 2, and the risk of bias (ROB) of studies is summarized in Table 2. Most of the included trials did not provide the details of their allocation concealment.

### 3.3. ASIA Motor Score

**Figure 3:**
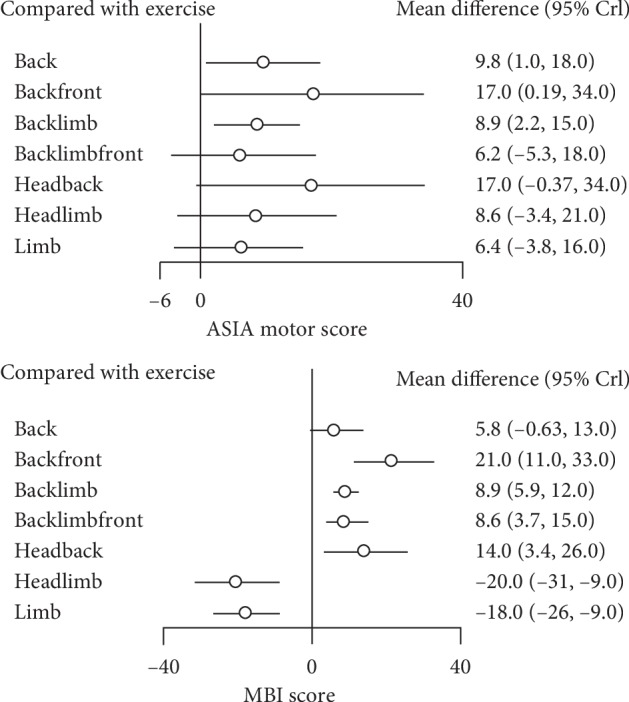
Forest plot of the mean difference between ASIA and MBI scales for each group.

Assessing primary outcome, the results of a meta-analysis suggested that exercise combined with EA on the back + front (SMD: 17; 95% CrI, 0.19∼34), back + limbs (SMD: 8.9; 95% CrI, 2.2∼15), head + back (SMD: 17; 95% CrI, 0.34∼34), back + limbs + front (SMD: 6.2; 95% CrI, –5.3∼18), head + limbs (SMD: 8.6; 95% CrI, –3.4∼21), back (SMD: 9.8; 95% CrI, 1.0∼18), and limbs (SMD: 6.4; 95% CrI, –3.8∼16) are superior to exercise without acupuncture. And, the therapies on the head + back (SMD: 17; 95% CrI, 0.34∼34) and the back + front (SMD: 17; 95% CrI, 0.19∼34) resulted in larger changes in the ASIA motor score than the other acupuncture therapies (see Figure 3).

**Figure 4:**
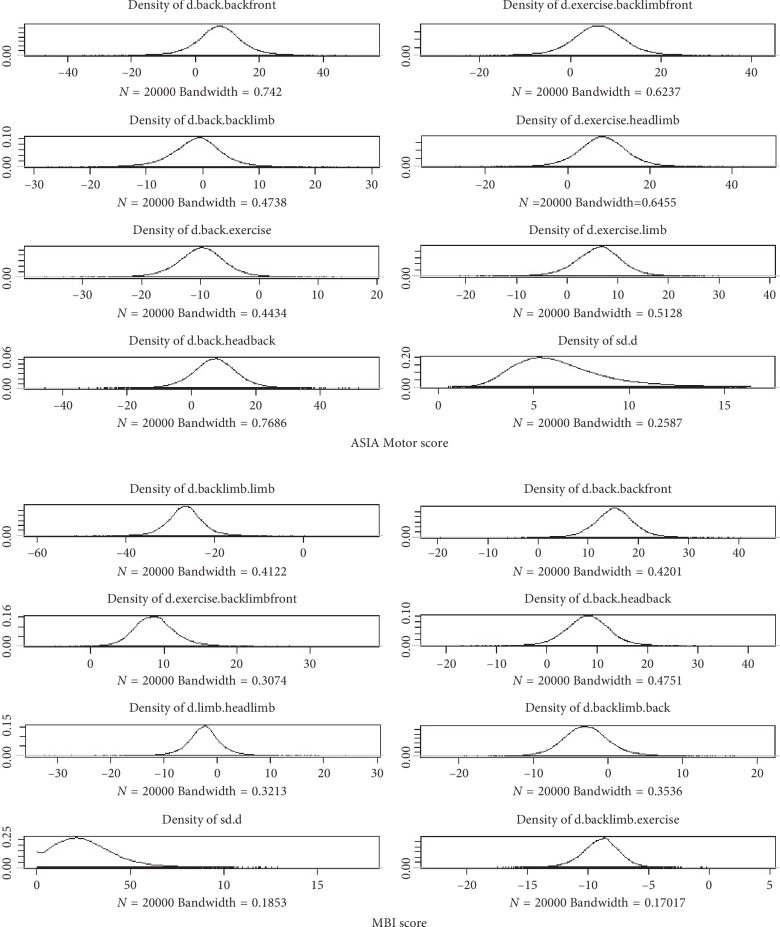
Probability density function of ASIA and MBI scales for each group.

The network meta-analysis showed that all therapies that combined exercise and acupuncture were more efficacious than exercise without acupuncture. The probability density function of the ASIA motor score consistency model is shown in Figure 4. *P* values for inconsistency tests are shown in Table 3.

### 3.4. MBI Score

The network meta-analysis indicated that exercise combined with EA on the back + front (SMD: 21; 95% CrI, 11∼33), back + limbs (SMD: 8.9; 95% CrI, 5.9∼12), head + back (SMD: 14; 95% CrI, 3.4–26), back + limbs + front (SMD: 8.6; 95% CrI, 3.7∼15), and back (SMD: 5.8; 95% CrI, –0.63∼13) were associated with a significantly higher improvement in the MBI score than exercise alone, but not head + limbs (SMD: –20; 95% CrI, –31∼9) and limbs (SMD: ∼18; 95% CrI, –26∼–9). And, the therapies on the back + front (SMD: 21; 95% CrI, 11∼33) resulted in the largest improvement in MBI score (see Figure 3). The probability density function of the MBI score consistency model is shown in Figure 4.

### 3.5. Safety

No serious adverse events (AE) were reported. And, most of the AE were moderate (e.g., postural hypotension, nausea, dizziness, and gastrointestinal complaints). Acupuncture was rarely associated with AEs (5.4%), which were generally mild (e.g., haematoma and nausea).

### 3.6. Sensitivity Analysis and Network Assumption

The heterogeneity in the pairwise meta-analysis was high in five comparisons: back vs. back + front, back vs. head + back, exercise vs. back + limb + front, exercise vs. head + limb, limbs vs. head + limbs; so, we chose the random effect model to cancel the statistical heterogeneity. As for clinical heterogeneity, meta regression was not done due to limited number of RCTs included in this article.

In total, 3 loops were in the network meta-analysis of the ASIA motor score, and 1 loop was in that of the MBI score. The pairwise meta-analysis and the network meta-analysis results did not significantly differ.

## 4. Discussion

The purpose of this network meta-analysis is to determine the effectiveness of various acupuncture therapies for SCI in improving motor function and daily living ability. Since rehabilitation training and neurotrophic drugs are combined for clinical routine treatment of SCI, routine rehabilitation training and medication are also included and analyzed as routine treatment. An assessment of the ROB indicates that most of the included studies were medium ROB. Preliminary analysis showed that except for head + limbs and limbs therapy, all other therapies improved patients' muscle strength and daily living ability comparing with conventional rehabilitation training (and drugs). The main results of the ranking chart showed that the acupuncture on the back + front, the back + head is most effective in improving muscle strength, followed by the back and back + limbs, head + limbs, limbs, back + front + limbs, and simple rehabilitation training (and drugs); while the back + front is most effective in improving the ability of daily life, followed by the back + head, back + limbs, back + front + limbs, back, rehabilitation training, limbs, and head + limbs. In addition, ancient mystical clinical devices or means may have a stronger placebo effect than drugs; therefore, acupuncture could be associated with greater placebo effects than drugs or rehabilitation. In terms of safety analysis, no serious adverse events related to acupuncture were reported in the selected studies, which may be associated with an overall low rate of serious AE in acupuncture. Longer time treatments appear to be more effective than short-term treatments. These results can be used as a reference for the efficacy and safety of clinical acupuncture in the treatment of SCI.

In short, the back + front, and the back + head strategy are the most effective ones. The best strategies would involve the acupoints in the back and the head is hardly a surprise, but what does the acupoints in the front have to do with the spinal cord, many would ask that question. The answer may lie in the way that acupuncturist sees the body. They see the body like a computer with keyboards or many buttons. Most of the time this computer runs on itself, but occasionally some error appears and it needs to be fixed. The acupuncturist knows where to push, and they use a needle to push it. Those buttons were called acupoints. We do not know who designed the computer or the keyboard, but some times the button that needs to be pushed lies not in the proximity of the illness or injury site, and may not even be linked to the injury site by the nerve system or the circulating system or any other systems, except by the meridians. There is currently not a satisfying explanation in modern science concerning this phenomenon. And, in the case of spinal cord injury, some of the buttons that needs to be pushed may lie in areas that is far away from the injured spinal cord. A skilled acupuncture finds such buttons by following the ancient ways. As for the deeper reasons for how and why these buttons work, may just be the real lesson that the modern medicine has to learn from Chinese traditional medicine.

Acupuncture or EA has not been used as a routine treatment for SCI, except in several Asian countries and regions. Acupuncture or EA can regulate immune function, such as cholinergic anti-inflammatory pathways, and participate in the inhibition of inflammatory response, while EA may promote nervous system regeneration by stimulating the nervous system. Most of the existing literature focused on acupuncture treatment of SCI complications such as neurogenic bladder, while less attention is paid to motor function or daily living ability. In contrast, acupuncture or EA treatment of SCI has a variety of specific methods, which only make it much more difficult to choose among various treatment methods.

To the authors' knowledge, all previous meta-analyses on acupuncture treatment of SCI laid emphasis on the effectiveness of acupuncture [6, 9, 35], but none recognized that acupuncture itself contains many different intervention strategies. This article analyzed the efficacy of acupuncture performed on different body parts to treat the same condition, SCI. And, judging from the results of this article, there are differences between these therapies.

Acupuncture and its derivative therapies mainly include needle acupuncture, moxibustion, EA, needle implantation, and even meridian massage. Even only within the category of needle acupuncture, there are differences such as the choosing of acupoints (usually depending on the meridian selection), depth, manipulation technique, stimulation time, frequency of treatment, and so on.

Admittedly, the categorizing strategy in this article is not the ideal way, but it is the first attempt to investigate the difference between many acupuncture therapies. Due to the limited number of relevant studies, this analysis has not been able to further classify and evaluate the selection of acupoints and meridians and their efficacy. We hope the number of related studies would increase in the future, and allow a more accurate classification and analysis of these acupuncture therapies.

The strengths of this study are as follows. We used Bayesian frameworks to compare various acupuncture therapies and rehabilitation (and medicine), and the results showed that acupuncture may be an effective and safe treatment, and there are differences between existing acupuncture therapies for SCI. And, its ranking chart may be useful for clinical practice and further research. In terms of data processing, we used scores at the end of treatment to arrive at clinically valuable conclusions.

## Figures and Tables

**Table 1:** Characteristics of included studies.

Author	Sample size and intervention		Duration of SCI	Outcome
Wong A. M.	50 patients, head + limbs + training; EA via the adhesive surface electrodes; bilateral Hou Hsi (SI3) and Shen Mo (B62); frequency, 75 Hz and pulse duration, 200 sec, 10 mV; each session was 30 min, five sessions per week	50 patients, training	58.6 ± 17.1 and 57.1 ± 18.7 days, respectively, in two groups	ASIA

Qin H. H.	22 patients, head + limbs + training; EA on upper 1/5 of parietal temporal anterior oblique (MS6), Piguan (ST31), Liangqiu (ST34), Zusanli (ST36), Fenglong (ST40), Taichong (LR3), Taixi (LI3), and Xuehai (SP10); each session was 20 mins, 100 Hz, once per day; total was 3 months	20, limbs + training 3 months	Not mentioned	ASIA, MBI

Wang S. J.	83, back + training; EA on Chongu, Dazhui, Taodao, Shenzhu, Shendao, Zhiyang, Jinsuo, Jizhong, Mingmen, Yaoyangguan, and Xiajishu; once per day for 3 months	83, training	31∼102 d	ASIA

Guo X. J.	52, back + limbs + training; EA above and below the injured segment, and Huantiao, Zusanli, Yanglingquan, Xuanzhong, Yinlingquan, and Sanyinjiao; 30 min per day, 30 days	52, limbs + training EA on Huantiao, Zusanli, Yanglingquan, Xuanzhong, Yinlingquan, and Sanyinjiao. 30 min per day, 30 days	4 months–4 years in the experiment, 5 months–4.5 years in control	ASIA, MBI,

Guan W.	43, back + limbs + training; EA on JIanyu, Binao, Quchi, Yangchi, Hegu, Piguan, Futu Zusanli, Fenglong, and Xuanzhong; 20 min per day, 30 days; vitamin B, acupoints injection of methylprednisolone near the injured segment, rehabilitation training for 30 days	43, training Vitamin B, acupoints injection of methylprednisolone near the injured segment, rehabilitation training. 30 days	2.72 ± 2.28 months in the experiment, 2.54 ± 2.43 months in control	ASIA, MBI

Jiang H. Y.	30, back + training; EA on bilateral Jiaji acupoints. 30 min per day, 80 days; rehabilitation training	30, training Rehabilitation training	3 h–14 d, average (3.1 ± 0.5) d in experiment. 3 h–15 d, average (3.0 ± 0.5) d in control	MBI

Wu K. X.	30, back + front + training; EA on bilateral Jiaji acupoints above and below the injured segment, 30 min per day, 7 days per week, 12 weeks; Zhongwan, Xiawan, Qihai, Guanyuan, Wailing, Huaroumen, Qipang, and Xiafengshi, 30 min per day, 3 days per week, 12 weeks	30, back + training EA on bilateral Jiaji acupoints above and below the injured segment, 30 min per day, 7 days per week, 12 weeks	(9.43 ± 7.26) months in experiment, (9.76 ± 6.98) months in control	ASIA, MBI

Ou Y. P.	42, back + front + limbs; EA on governor meridian and Jiaji points and Jianyu, Quchi, Waiguan, Hegu, Huantiao, Fengshi, Yanglingquan, Jiegu, Taichong, Shenshu, Pangguanshu, Ciliao, Zhongji, Tianshu, Chengshan, Jiexi, Dachangshu, and Changqiang; 30 min per day, 5 times a week, total of 8 weeks; rehabilitation training such as muscle training, balance, and walking	42, training Rehabilitation training such as muscle training, balance, and walking	17–73 d in experiment, 21–70 d in control	MBI

Li X. N.	20, back + limbs + training; EA on Jiaji acupoints, Dazhui, Mingmen, Yanglingquan, Zusanli, Shenyu, and Huiyang; 1～2 Hz. 30 min per day	20, back + training EA only on Jiaji acupoints. Rehabilitation training	Not mentioned	ASIA, MBI
Deng C.	20, back + medicine; EA on Zhibian, Huantiao, Piguan, Futu, Yanglingquan, Zusanli, and Kunln; each treatment session was 30 min, 5 times per week, 8 weeks; monosialotetrahexosyl ganglioside sodium 20 mg, iv.qd	20, medicine Monosialotetrahexosyl ganglioside sodium 20 mg, iv.qd	1–6 months	ASIA

Deng N.	12, back + limbs + training; EA on governor meridian and Jiaji points above and below the injured site, and Chongmen, Piguan, Huantiao, Weizhong, Zhibian, and Yanglingquan; 2–100 Hz. Each session was 30 min, once per day, 5 times a week, total of 8 weeks	12, training Rehabilitation training	(81.501 ± 6.29) d in experiment, (78.17 ± 6.25) d in control	ASIA

Qian Y.	38, limbs + training; EA on Shangxing, Shenting, Shenzhu, Baihui, Jizhong, Lingtai, Yaoyangguan, Xuanshu, and Yaoshu; one session was 30 min, 6 times per week, 4 weeks; rehabilitation training	38, training Rehabilitation training and tizanidine hydrochloride tablets	Not mentioned	ASIA

Sun S. B.	20, back + front + limbs + training; Baihui (GV 20), Fengfu (GV 16), Dazhui (GV 14), Zhiyang (GV 9), Mingmen (GV 4), and Yaoyangguan (GV 3) were selected as the main acupoints; Zhongji (CV 3), Guanyuan (CV 4), Qihai (CV 6), and Guilai (ST 29) were additionally selected for the patients with fecal and urinary incontinence or urinary retention; Jianyu (LI 15), Jianliao (TE 14), Quchi (LI 11), and Hegu (LI 4) were additionally selected for the patients with upper limb dysfunction; Zusanli (ST 36), Xuehai (SP 10), Yanglingquan (GB 34), and Sanyinjiao (SP 6) were additionally selected for the patients with lower limb dysfunction; once a day, 6 times a week, and 12 weeks in total; Conventional rehabilitation training: joint rehabilitation training, exercise rehabilitation training, respiratory training, bladder rehabilitation training, and intestine tract rehabilitation training; once a day, 6 times a week, and 12 weeks in total	20, training Conventional rehabilitation training: joint rehabilitation training, exercise rehabilitation training, respiratory training, bladder rehabilitation training, and intestine tract rehabilitation training. Once a day, 6 times a week, and 12 weeks in total	2.3–5.4 months in experiment, 2.4–5.2 months in control	ASIA

Huo H. X.	20, head + back + training; EA on Jiaji points above and below the injured site, and upper 1/5 of parietal temporal anterior oblique (MS6); 30 min per day, 6 days per week, 8 weeks in total; traditional rehabilitation training	20, back + training EA on Jiaji points above and below the injured site. 30 min per day, 6 days per week, and 8 weeks in total. Traditional rehabilitation training	1–3 months	ASIA, MBI
Xiao H.	30, back + front + limbs + training; acupuncture on 3 cun above and 2 cun below CV8 of KI and ST meridian in the abdomen, and on the Jiaji points of 3 vertebra segments above and 2 vertebra segments below the injured segment on the back, with the additional points on the limbs bilaterally; acupoints changed every other day on the back and abdomen; 30 mins per day for 3 months; rehabilitation includes strength training, wheelchair driver training, transfer and gait training, practical gait training, and so on	30, training Traditional rehabilitation training	Not mentioned	MBI

Xie J. D.	43, back + limbs + training; EA on Azhui, Lingtai, Mingmen, Yaoyang, Shousanli, Quchi, Waiguan, Zusanli, Sanyinjiao, Taixi, Yanglingquan, Pangguangshu, Guanyuan, Qihai; 30 min per day, 10 days a session, and followed by a 5-day rest. 6 sessions in total; rehabilitation training	43, training Rehabilitation training	Not mentioned	MBI

Guo Y. H.	44, back + limbs + training; EA on Dazhui, Lingtai, Mingmen, Yaoyang, Quchi, Waiguan, Hegu, Shousanli, Zusanli, Sanyinjiao, Taixi, Futu, Yanglingquan, Guanyuan, Qihai, Pangguangshu; once per day, each treatment session was 30 min, 10 days a session, and followed by a 5-day rest. 3–5 sessions; rehabilitation training	44, training Rehabilitation training	Not mentioned	MBI

Liang T. Y.	40, back + front + limbs + training; EA on Dazhui, Mingmen, Lingtai, Yaoyang, Quchi, Waiguan, Shousanli, Taixi, Yanglingquan, Sanyinjiao, Zusanli, Guanyuan, Qihai, Pangguangshu; once per day, each treatment session was 30 min, 10 days a session, and followed by a 5-day rest; 6 sessions in total; rehabilitation training.	40, training Rehabilitation training	Not mentioned	MBI

Du Y. P.	36, back + limbs + training; EA on Dazhui, Mingmen, Guanyuan, Qihai, Pangguangshu, Lingtai, Yaoyang, Quchi, Waiguan, Shousanli, Taixi, Yanglingquan, Sanyinjiao, Zusanli; once per day, 30 min, 10 days a session, and followed by a 5-day rest. 6 sessions in total; rehabilitation training	36, training Traditional rehabilitation training	Not mentioned	MBI

Chen D.	20, back + limbs + training. EA on governor meridian and bladder meridian on acupoints above and below the injured site, and acupoints on three yin meridian and three yang meridian; traditional rehabilitation training	20, training Traditional rehabilitation training.	Not mentioned	ASIA, MBI
Guo J.	50, back + limbs + training; EA on Jiaji points and Dazhui, Mingmen, Zusanli, Yanglingquan, Huiyang, Shenshu; each session was 30 min, once per day, 6 times a week, a total of 8 weeks	50, training and medicine Rehabilitation training, herbal steam	(24.6 ± 3 1) d in experiment, (24 5 ± 3.2) d in control	ASIA, MBI

Zhao L. S.	86, back + front + limbs + training; EA on Shenshu, Mingmen, Guanyuan, Taixi, Zusanli, Pishu; each session was 30 min, once per day, 5 times a week, a total of 4 weeks	86, training	Not mentioned	ASIA

ASIA: American Spinal Injury Association. MBI: Modified Barthel Index.

**Table 2:** Risk of bias assessment (ROB).

Author	Random sequence generation	Allocation concealment	Blinding of outcome assessment	Incomplete outcome data	Selective reporting
Qin H. H. [26]	L	U	U	L	L
Wang S. J. [17]	L	H	U	L	U
Guo X. J. [23]	L	U	U	L	L
Guan W. [21]	L	H	L	L	U
Jiang H. Y. [30]	L	H	L	L	U
Wu K. X. [27]	U	H	U	L	L
Ou Y. P. [32]	L	U	U	L	U
Li X. N. [25]	L	U	U	L	L
Deng N. [20]	L	H	L	L	U
Deng C. [14]	L	U	U	L	U
Qian Y. [15]	L	U	U	L	U
Sun S. B. [16]	U	H	U	L	U
Huo H. X. [24]	U	H	L	L	L
Xiao H. [34]	U	H	L	L	L
Xie J. D. [33]	U	U	U	L	L
Guo Y. H. [29]	L	H	U	L	L
Liang T. Y. [31]	L	H	L	L	L
Du Y. P. [28]	L	U	L	L	L
Chen D. [19]	L	U	U	U	L
Guo J. [22]	L	H	U	U	L
Zhao L. S. [18]	L	U	U	U	L
Wong A. M. [12]	L	L	H	L	L

L: low risk of bias; H: high risk of bias; U: unclear. Risk of bias assessment.

**Table 3:** *P* values for inconsistency tests of the ASIA motor score and MBI score.

Group 1	Group 2	*P* (ASIA)	*P* (MBI)
Back	Back + front	NA	NA
Back	Back + limb	0.62654592	0.385499
Back	Exercise	0.59531188	0.2816815
Back	Head + back	NA	NA
Back + limb	Exercise	0.10864181	0.1184854
Back + limb	Limb	0.06084375	NA
Back + limb + front	Exercise	NA	NA
Exercise	Head + limb	0.35802045	NA
Exercise	Limb	0.35561283	NA
Head + limb	Limb	0.396328	NA

## Data Availability

The data used to support the findings of this study are available from the corresponding author upon request.

## Supplementary Materials

Supplementary Materials Statistical code of R software.
