# Supplementary material for: The Effect of Different Acupuncture Therapies on Neurological Recovery in Spinal Cord Injury: A Systematic Review and Network Meta-Analysis of Randomized Controlled Trials
Source: Evid Based Complement Alternat Med. 2019 Oct 27;2019:2371084. doi: 10.1155/2019/2371084 (PMC6854954; doi:10.1155/2019/2371084)
Supplement: Supplementary Materials — Statistical code of R software. [file 2371084.f1.docx]

**Supplement 1 : Statistical code**

The data was first inputted using the gemtc software. Then was analyzed using the R software. The code of R software is as follows:

**#ASIA motor score**

rm(list=ls())

getwd()

setwd ("E:\\acpmeta")

library(lattice)

library(coda)

library(codetools)

library(testthat)

library(gemtc)

library(R2OpenBUGS)

library(igraph)

library(XML)

library(rjags)

library(R2jags)

library(runjags)

library(BRugs)

library(R2WinBUGS)

network<-read.mtc.network("E:/acpmeta/sciacp.gemtc")

print(network)

model<- mtc.model(network,type = "consistency",n.chain = 3)

tiff(file="0101hetero.tiff")

result.anohe<-mtc.anohe(network,n.adapt=5000,n.iter=20000,thin=1)

summary(result.anohe)

plot(result.anohe)

summary.anohe<-summary(result.anohe)

plot(summary.anohe,xlim=log(c(0.1,14)))

dev.off()

results<-mtc.run(model,sampler="JAGS",n.adapt=5000,n.iter=20000,thin=1)

summary(network)

summary(results)

tiff(file="0101forest.tiff")

forest(results)

dev.off()

summary(relative.effect(results, "excercise", c("back", "backfront", "backlimbs", "backlimbsfront", "headback", "headlimbs","limbs")))

plot(network)

tiff(file="0101gelman.tiff")

gelman.plot(results)

dev.off()

tiff(file="0101density.tiff")

plot(results)

dev.off()

ranks<-rank.probability(results,preferredDirection=1)

print(ranks)

tiff(file="0101ranks.tiff")

plot(ranks)

plot(ranks,beside=TRUE)

dev.off()

mtc.nodesplit.comparisons(network)

result<-mtc.nodesplit(network)

summary(result)

tiff(file=" 0101NodesplitForest.tiff")

plot(summary(result))

dev.off()

**#MBI score**

rm(list=ls())

getwd()

setwd ("E:\\acpmeta")

library(lattice)

library(coda)

library(codetools)

library(testthat)

library(gemtc)

library(R2OpenBUGS)

library(igraph)

library(XML)

library(rjags)

library(R2jags)

library(runjags)

library(BRugs)

library(R2WinBUGS)

network<-read.mtc.network("E:/acpmeta/ MBIsciacp.gemtc")

tiff(file="0102hetero.tiff")

result.anohe<-mtc.anohe(network,n.adapt=5000,n.iter=20000,thin=1)

summary(result.anohe)

plot(result.anohe)

summary.anohe<-summary(result.anohe)

plot(summary.anohe,xlim=log(c(0.1,14)))

dev.off()

print(network)

model<- mtc.model(network,type = "consistency",n.chain = 3)

results<-mtc.run(model,sampler="JAGS",n.adapt=5000,n.iter=20000,thin=1)

summary(network)

summary(results)

tiff(file="0102forest.tiff")

forest(results)

dev.off()

summary(relative.effect(results, "excercise", c("back", "backfront", "backlimbs", "backlimbsfront", "headback", "headlimbs","limbs")))

plot(network)

tiff(file="0102gelman.tiff")

gelman.plot(results)

dev.off()

tiff(file="0102density.tiff")

plot(results)

dev.off()

ranks<-rank.probability(results,preferredDirection=1)

print(ranks)

tiff(file="0102ranks.tiff")

plot(ranks)

plot(ranks,beside=TRUE)

dev.off()

mtc.nodesplit.comparisons(network)

result<-mtc.nodesplit(network)

summary(result)

tiff(file=" 0102NodesplitForest.tiff")

plot(summary(result))

dev.off()
